# Evaluation of the Efficacy of a Recombinant Adenovirus Expressing the Spike Protein of Porcine Epidemic Diarrhea Virus in Pigs

**DOI:** 10.1155/2019/8530273

**Published:** 2019-10-08

**Authors:** Xinsheng Liu, Donghong Zhao, Peng Zhou, Yongguang Zhang, Yonglu Wang

**Affiliations:** ^1^State Key Laboratory of Veterinary Etiological Biology, Key Laboratory of Animal Virology of Ministry of Agriculture, Lanzhou Veterinary Research Institute, Chinese Academy of Agricultural Sciences, Lanzhou 730046, China; ^2^Jiangsu Co-Innovation Center for Prevention and Control of Important Animal Infectious Diseases and Zoonoses, Yangzhou 225009, China

## Abstract

In recent years, many studies have shown that recombinant adenovirus live vector-based vaccines are a promising novel vaccine candidate against virus infection. Therefore, in this study, a new type of recombinant adenovirus expressing the spike (S) protein of porcine epidemic diarrhea virus (PEDV), rAd-PEDV-S, was generated, and its characteristics were determined. Then, its efficacy as a vaccine candidate was evaluated in 4-week-old pigs. The results showed that the S protein could be well expressed at a high level in rAd-PEDV-S-infected cells and that the viral titers could reach 10^11^ PFU/mL. Further animal experimental results showed that rAd-PEDV-S elicited a significant PEDV-specific humoral immune response after vaccination (*P* < 0.05). In addition, rAd-PEDV-S provided partial protection for pigs against the highly virulent PEDV challenge. The results presented in this study indicate that the adenovirus vector can be used as a vaccine delivery vector for the development of a PEDV vaccine and is a promising novel vaccine candidate for future prevention and control of porcine epidemic diarrhea (PED), but its efficacy still needs to be improved in the future.

## 1. Introduction

Porcine epidemic diarrhea virus (PEDV), a member of the order Nidovirales, family Coronaviridae, and genus *Alphacoronavirus*, is the causative agent of porcine epidemic diarrhea (PED), which is characterized by watery diarrhea, vomiting, dehydration, growth retardation, and high morbidity and mortality in suckling piglets [1, 2]. PEDV is an enveloped, single-stranded, positive-sense RNA virus, and its genome is approximately 28 kb in length and is composed of seven open reading frames (ORF1a, ORF1b, and ORF2–6) [3, 4]. Four structural proteins, namely, the spike (S) glycoprotein, membrane (M) protein, envelope (E) protein, and nucleocapsid (N) protein, and one accessory protein, ORF3, are encoded by ORF2–6 [4, 5]. Among the structural proteins, the S protein is the major envelope glycoprotein responsible for receptor binding and cell membrane fusion and entry [6, 7]. Therefore, the S protein is considered the main target for neutralizing antibodies against PEDV and has been frequently used in phylogenetic analysis, establishment of serologic diagnostic methods, and development of new vaccines.

Since the PEDV outbreaks occurred in Asian swine herds in the 1980s, the disease has become widespread and ultimately endemic in the region [4, 8]. Furthermore, new highly virulent PEDV strains emerged in China at the end of 2010 and caused large economic losses [9, 10]. Starting in the spring of 2013, the PEDV variant outbreaks were successively reported in the United States (US) and subsequently spread to other countries in North America and Europe [5, 11–13]. Although attenuated and inactivated PEDV vaccines have been developed and widely used in Asia for many years, severe PEDV outbreaks have still been reported in recent years [14–16]. The main reason for this result is that vaccines based on European and other classical PEDV strains failed to control the more recent virulent PEDV strains in Asia [5, 14]. Therefore, it is necessary and urgent to develop new PEDV vaccines from highly virulent variants of the virus.

At present, all commercially available vaccines, especially in Asia, are traditional live attenuated or inactivated/killed vaccines, and their efficacy needs to be further developed and improved through further study [11, 17, 18]. Therefore, many studies have been carried out for the development of next-generation PEDV vaccines based on the S protein, such as a recombinant *Lactobacillus* vaccine [19], a recombinant DNA plasmid vaccine [20], an attenuated *Salmonella typhimurium* vaccine [21], a recombinant *Parapoxvirus* vaccine [22], a recombinant orf virus (ORFV) vaccine [23], and a recombinant vesicular stomatitis virus (VSV) vaccine [24]. Moreover, the first commercial PEDV vaccine used in the US, called the iPED vaccine, was based on a truncated version of the PEDV spike gene and a pVEK replicon vector derived from the Venezuelan equine encephalitis virus [14]. The vaccine induced immunity in young pigs after two doses given intramuscularly (IM) in a three-week interval, significantly reduced the severity of clinical signs (diarrhea), and reduced viral shedding for the first 72 hours [14]. Going forward, new genetically engineered vaccines will be an important direction for the development of PEDV in the future.

Currently, adenovirus vector systems derived from human adenoviruses have been widely used in the development of new-generation genetically engineered vaccines [25–27]. They show more advantages than other vectors, such as easy construction, high efficiency for gene transfer and propagation, high titers and safety, and strong induction of humoral, mucosal, and cellular immune responses [25]. Therefore, the recombinant adenovirus vector vaccine is a promising approach for the development of new viral vaccines. However, so far, toward a promising vaccine, the study of a recombinant adenovirus vector vaccine for PEDV is underdeveloped. Therefore, the development of new PEDV vaccines based on recombinant adenovirus vectors is needed. To achieve this objective, in this study, a novel recombinant adenovirus expressing the PEDV S protein was generated, and its immunogenicity and protective efficacy were evaluated in 4-week-old pigs.

## 2. Materials and Methods

### 2.1. Ethics Statement

This study was approved by the Institutional Animal Use and Care Committee of Lanzhou Veterinary Research Institute of the CAAS. All piglets used in the present study were humanely maintained in a biosafety laboratory during the experiment and euthanized at the end of the experiment according to the animal care and use protocols of the institute.

### 2.2. Virus and Cells

The highly virulent Chinese genotype GIIa PEDV strain CH/HBXT/2018 (GenBank accession number MH816969) was isolated by our laboratory and propagated in Vero cells (ATCC CCL-81) as previously described [28]. The Ad5Max adenovirus vector system derived from human adenovirus serotype 5 was obtained from GeneCreate Biological Engineering Company (Wuhan, China). The Vero cells were grown in Dulbecco's modified Eagle's medium (DMEM; Invitrogen, USA) containing 10% heat-inactivated fetal bovine serum (FBS; Invitrogen, Australia) and 1% antibiotics and antimycotics (10,000 units of penicillin, 10,000 *μ*g of streptomycin, and 25 *μ*g of Fungizone® per milliliter) (Gibco™, USA) and cultured at 37°C with 5% CO_2_. HEK293A cells were obtained from GeneCreate Biological Engineering Company (Wuhan, China) and were cultured in DMEM (Invitrogen, USA) with 10% heat-inactivated FBS (Gibco™, USA).

### 2.3. Construction of Recombinant Transfer Plasmid

The complete sequence of the S gene of the PEDV strain CH/HBXT/2018 was amplified and sequenced. Then, restriction enzyme sites for *Not* I and *Afl* II were introduced separately at the 5′ and 3′ ends, respectively, of the S gene along with a His-tag and synthesized by Sangon Biotech (Shanghai, China). The synthesized S gene products encoding the S protein and the pDC316-mCMV-EGFP vector (Invitrogen, USA) were digested with *Not* I and *Afl* II and then ligated using T4 DNA Ligase (NEB, USA) at 16°C for 1 hour. The ligation products were transformed into TOP10 competent cells (Invitrogen, USA), and positive clones were confirmed by restriction enzyme digestion and sequencing. The correct recombinant transfer bacmid was named “pDC316-S-EGFP.”

### 2.4. Generation and Characterization of the Adenovirus-PEDV-S Recombinant Virus

HEK293A cells were seeded in a 6 cm dish (Corning, USA), placed in a 37°C incubator with 5% CO_2_ until a 70–80% confluent cell monolayer formed, and then transfected with 1.5 *μ*g of the transfer bacmid pDC316-S-EGFP and 6 *μ*g of pBHGlox(delta)E1,3 using the Lipofectamine™ 2000 transfection reagent (Invitrogen, USA). After incubation for 6–10 hours at 37°C with 5% CO_2_, the fresh growth medium (containing DMEM and 10% heat-inactivated FBS) was added, and the cell culture continued at 37°C and 5% CO_2_, with daily observation for cytopathic effects (CPEs). When more than 90% of cells showed CPEs, the cell cultures were harvested, frozen, and thawed three times and centrifuged for the next passage of the adenovirus-PEDV-S recombinant virus (rAd-PEDV-S). After three passages, the third passage (P3) rAd-PEDV-S was purified by cesium chloride gradient centrifugation, and the titers of the recombinant adenoviruses were tested by fluorescence dilution as follows: 1 × 10^4^ HEK293A cells were seeded into 96-well plates and then maintained at 37°C in a humidified 5% CO_2_ incubator for 24 hours. The harvested recombinant adenoviruses were 10-fold serially diluted in DMEM (Invitrogen, USA) (from 10^−1^ to 10^−11^), mixed well, and vortexed. After confluence, the monolayer cells were washed with PBS (Gibco™, USA). Then, 100 *μ*l of 10-fold serially diluted virus suspensions was inoculated in two replicates per dilution. After 24 hours of incubation, 100 *μ*l of DMEM (Invitrogen, USA) with 10% heat-inactivated FBS (Gibco™, USA) was added. Cells were monitored for 72 hours, and the viral titers were determined using a fluorescence microscope. Furthermore, the expression level of the PEDV S gene in rAd-PEDV-S was measured by real-time fluorescence quantitative RT-PCR according to the method used in the previous study [29].

### 2.5. Detection of S Gene Expression by Western Blot

P3 rAd-PEDV-S was used to infect HEK293A cells, and the cells were harvested after 120 hours. The cells were lysed using the lysis buffer (Thermo Scientific, Waltham, MA) containing protease inhibitors (Invitrogen, USA), frozen/thawed 3 times, and then centrifuged and collected at 4°C and 12000 rpm for 10 minutes. Then, the proteins were separated by SDS-PAGE and transferred to nitrocellulose membranes under a constant voltage of 80 V for 2 hours. The blots were blocked with 5% skim milk at 37°C for 1 hour and then incubated with a primary mouse anti-His-tag monoclonal antibody (mAb) (1 : 1000 dilution; Abcam, USA) for 1 hour at 37°C. After washing 3 times for 5 minutes per wash with TBS-Tween 20 (50 mM Tris, 150 mM NaCl, and 0.05% Tween 20, pH 7.6), the membrane was incubated with a secondary horseradish peroxidase- (HRP-) labeled goat anti-mouse IgG antibody (1 : 2000 dilution; Abcam, USA) for 1 hour at 37°C. Finally, the membrane was washed and visualized using an ECL chemiluminescent substrate reagent kit (Thermo Scientific, USA).

### 2.6. Pig Immunization and Challenge Experiment

Twenty 4-week-old pigs that were tested seronegative for PEDV by a commercial enzyme-linked immunosorbent assay (ELISA) kit (Biovet, Canada) were obtained from a commercial pig farm with no previous herd history of PED outbreak or PEDV vaccination. All of the pigs were raised in the laboratory animal facility at the Lanzhou Veterinary Research Institute, and each group of pigs was housed in a different room. The vaccination doses were determined based on previous published studies of the PEDV S gene-based recombinant virus vaccine [22–24]. As shown in Table 1, all piglets were randomly divided into four groups (G1–G4) each with five pigs. Groups 1–3 were immunized IM with 10^10^ PFU/mL Ad-blank, 10^10^ PFU/mL rAd-PEDV-S, and 2 mL of the commercial PEDV inactivated vaccine (inactivated virus of a genotype GIIb strain; virus titer before inactivation ≥ 10^7^ TCID_50_). Group 4 was immunized IM with phosphate-buffered saline (PBS) as a mock control. All pigs were first immunized at 0 days post vaccination (dpv) and received booster immunizations at 21 dpv under the same conditions. Serum samples from all groups were collected at 0, 7, 14, 21, and 28 dpv for antibodies.

At 28 dpv, to evaluate the efficacy of rAd-PEDV-S, all pigs of each group were challenged orally with 2 mL of cell-cultured P4 virus containing 1 × 10^5^ TCID_50_ highly virulent genotype GIIb PEDV strain CH/HNPJ/2017 [28]. Before the challenge, fecal rectal swabs were collected from all piglets and tested by real-time PCR to confirm PEDV-negative results. After the challenge, clinical signs of PEDV infection were observed daily for 7 days, and clinical scores of fecal consistency were determined according to the methods of a previous study as follows: 0 = normal, 1 = pasty, 2 = semiliquid, and 3 = liquid [30]. Fecal samples were collected daily to monitor virus shedding in feces during the experiment period.

### 2.7. ELISA

An indirect ELISA based on a PEDV S2 protein (approximately 57 kDa), which encodes the 790–1386 aa spike protein of the genotype GII PEDV strain and was expressed as a recombinant protein in *Escherichia coli* by our laboratory, was developed to assess IgG and IgA antibody responses in pigs immunized with rAd-PEDV-S. In brief, a concentration of 0.1 *μ*g/mL purified PEDV S2 protein in 0.05 M NaHCO_3_ was used to coat ELISA plates (Corning, USA) and then incubated with samples (1 : 200 dilution) at 37°C for 1 hour. After the plate was washed with PBST 3 times, an HRP-conjugated goat anti-pig IgG mAb (1 : 10000 dilution; Abcam, USA) or HRP-conjugated goat anti-pig IgA mAb (1 : 10000 dilution; Abcam, USA) was added and incubated at 37°C for 0.5 hours. Then, the plate was washed with PBST 3 times, followed by the addition of the 3,3′,5,5′-tetramethylbenzidine (TMB) substrate and incubation at 37°C for 15 minutes. Then, 50 *μ*L of stop solution was added, and the absorbance was determined at 450 nm.

### 2.8. Virus Neutralization (VN) Test

Neutralizing antibody responses elicited by immunization with rAd-PEDV-S were assessed by the virus neutralization (VN) test as described previously [17]. Neutralizing antibody titers were calculated as the reciprocal of the highest serum dilution that inhibited CPEs.

### 2.9. Viral RNA Extraction and Real-Time PCR

Viral RNA was extracted from intestinal contents using the RNeasy Mini Kit (Qiagen, USA) according to the manufacturer's instructions. Real-time PCR targeting the PEDV N gene was performed as described previously [17].

### 2.10. Statistical Analysis

Statistical analysis was performed using the SPSS 16 software. Statistical significance among different experimental groups was determined using one-way ANOVA with Tukey's multiple-comparison test. A difference was considered significant when the *P* value was less than 0.05.

## 3. Results

### 3.1. Generation and Characterization of the Recombinant Virus rAd-PEDV-S

**Figure 1:**
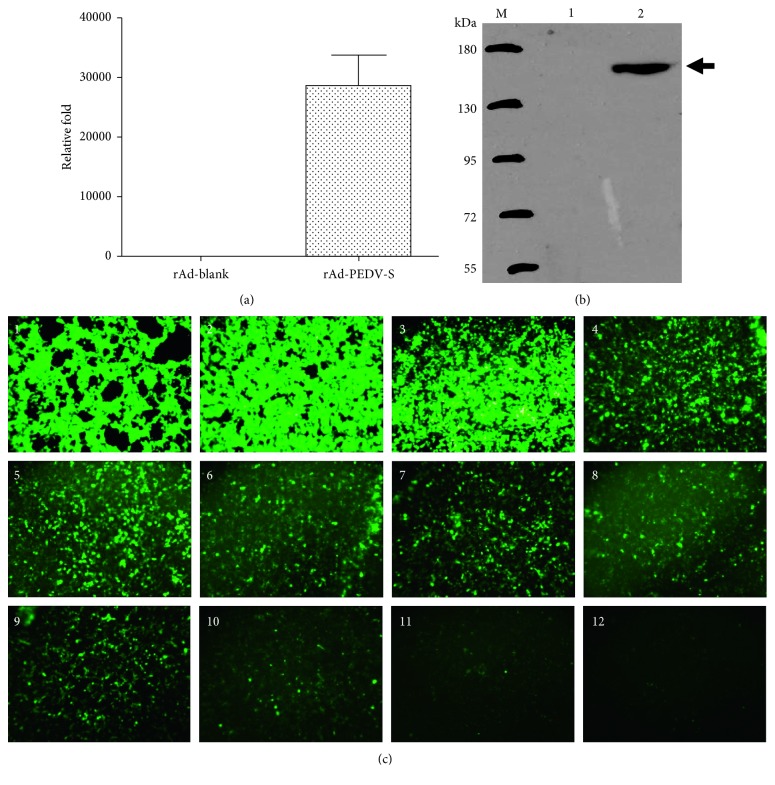
Generation and characterization of the recombinant rAd-PEDV-S virus. (a) Measurement of expression levels of the recombinant virus rAd-PEDV-S in infected HEK293A cells. Noninfected HEK293A cells were used as a negative control. (b) Western blot demonstrating the expression of full-length PEDV S (approximately 153 kDa) by the recombinant virus rAd-PEDV-S in the cell culture in vitro. Cell lysates from cells infected with wild-type adenovirus were used as negative controls. The blot was developed with an anti-His-tag mAb. (c) Titer of the recombinant virus determined by the rAd-PEDV-S fluorescence dilution assay. Green fluorescence was emitted by the enhanced green fluorescent protein (EGFP) derived from the vector. The results showed that the recombinant virus rAd-PEDV-S could be well replicated in HEK293A cells and that its titers could reach 10^11^ PFU/ml.

The S protein gene of the highly virulent Chinese genotype GIIa PEDV strain CH/HBXT/2018 was amplified and sequenced, and the length of the complete coding sequence was 4158 bp, corresponding to 1386 aa. The synthesized S protein gene containing restriction enzyme sites and a His-tag was confirmed by sequencing (data not shown). The recombinant transfer bacmid pDC316-S-EGFP was confirmed by restriction enzyme digestion and sequencing (data not shown). The expression levels of the recombinant virus rAd-PEDV-S were assessed in vitro by real-time fluorescence quantitative RT-PCR, and the results showed that the PEDV S gene was well expressed at high levels (Figure 1(a)). Expression of PEDV S by the recombinant virus rAd-PEDV-S was further assessed by western blot assay, and the results revealed 153 kDa protein bands, consistent with the predicted size of the S protein, in HEK293A cells transfected with rAd-PEDV-S (Figure 1(b)). No specific protein bands were observed in HEK293A cells infected with Ad-blank alone (Figure 1(b)). The results of the fluorescence dilution assay also showed that the recombinant virus rAd-PEDV-S could be well replicated in HEK293A cells and that its titers could reach 10^11^ PFU/mL (Figure 1(c)).

### 3.2. Antibody Responses against PEDV in Vaccinated Pigs

**Figure 2:**
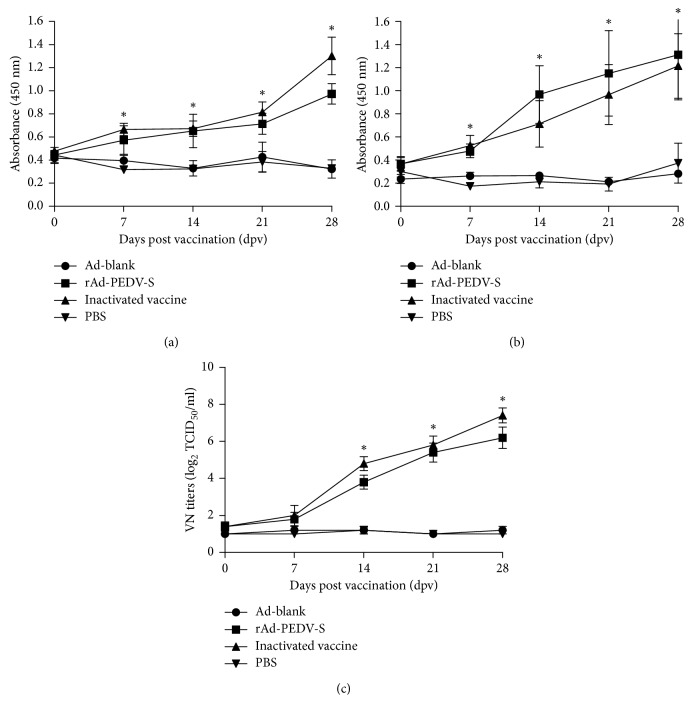
Antibody responses against PEDV vaccination from serum samples. Pigs were vaccinated at 0 dpv and then challenged orally with 2 mL of diluted virus containing 1 × 10^5^ TCID_50_ highly virulent genotype GIIb PEDV strain CH/HNPJ/2017 at 28 dpv. Serum samples were collected at 0, 7, 14, 21, and 28 dpv. (a) PEDV-specific IgG antibody responses against PEDV from serum samples from vaccinated pigs determined using an indirect ELISA. (b) PEDV-specific IgA antibody responses against PEDV from serum samples from vaccinated pigs determined using an indirect ELISA. (c) PEDV-specific VN antibody responses elicited by vaccination with the recombinant virus rAd-PEDV-S. Neutralizing antibody titers were calculated as the reciprocal of the highest serum dilution that inhibited CPEs. Error bars represent the SEM. Statistical significance among different experimental groups was determined using one-way ANOVA with Tukey's multiple-comparison test. The asterisk indicates a significant difference, with the *P* value less than 0.05.

Anti-PEDV-specific IgG and IgA antibody titers of different groups induced by the recombinant virus rAd-PEDV-S were evaluated in pigs following immunizations IM from 0 dpv to 28 dpv by an indirect ELISA based on a PEDV S2 protein. As shown in Figure 2(a), compared with the Ad-blank and PBS groups, the rAd-PEDV-S group and commercial PEDV inactivated vaccine group exhibited detectable specific IgG antibody titers initially at 7 dpv that rose continuously until 28 dpv (*P* < 0.05). At 7 dpv, 14 dpv, 21 dpv, and 28 dpv, the specific IgG antibody titers of the rAd-PEDV-S group and the commercial vaccine group were higher than those of the Ad-blank and PBS groups (*P* < 0.05). Meanwhile, the specific IgG antibody titer of the commercial inactivated vaccine group was slightly higher than that of the rAd-PEDV-S group at 7, 21, and 28 dpv, but there was no significant difference between them (*P* > 0.05).

Similarly, as shown in Figure 2(b), the specific IgA antibody titers of pigs from the recombinant virus rAd-PEDV-S and commercial inactivated vaccine groups were significantly higher than those of pigs from the Ad-blank and PBS groups from 7 to 28 dpv (*P* < 0.05), while there was no significant difference between the rAd-PEDV-S and inactivated vaccine groups (*P* > 0.05).

The ability of the recombinant virus rAd-PEDV-S to induce VN antibodies against PEDV was assessed using a VN test. Similar to the levels of serological IgG and IgA antibodies, the results of the VN test revealed that immunization IM with rAd-PEDV-S also elicited VN responses against PEDV in all vaccinated pigs (Figure 2(c)). Meanwhile, no VN responses were detected in the pigs of the Ad-blank and PBS groups (Figure 2(c)). Together, these results indicated that the recombinant virus rAd-PEDV-S could induce a strong humoral immune response in pigs.

### 3.3. Protective Effect of the Recombinant Virus rAd-PEDV-S as a Vaccine Candidate

To assess the protective effect of the recombinant virus rAd-PEDV-S, pigs from all four groups (Table 1) were challenged orally with 1 × 10^5^ TCID_50_ highly virulent genotype GIIb PEDV strain CH/HNPJ/2017 at 28 dpv. All four groups of pigs were apparently healthy and had no clinical symptoms before the oral challenge. Fecal rectal swabs were collected from all piglets at 0 days post inoculation (dpi), and the results of real-time PCR confirmed that all piglets in each group were PEDV negative (Table 2). At 1 dpi, typical clinical signs of PEDV infection were observed in 1/5 pigs from the Ad-blank group and in 2/5 pigs from the PBS group, while no pigs from rAd-PEDV-S and inactivated vaccine groups developed clinical signs of PED (Table 2). All pigs (5/5) from the Ad-blank and PBS groups developed severe diarrhea at 3 and 2 dpi, respectively, and then were euthanized after infection was confirmed by clinical symptoms and fecal viral shedding (Table 2). Pigs in the rAd-PEDV-S and inactivated vaccine groups were PEDV positive and developed clinical signs of PED at 4 dpi (Table 2). Finally, in the rAd-PEDV-S and inactivated vaccine groups, 2/5 and 3/5 of pigs, respectively, were protected during the observation period. Notably, compared with the Ad-blank and PBS groups, the rAd-PEDV-S and inactivated vaccine groups displayed obviously delayed virus shedding and clinical signs of PED in pigs (Table 2). Therefore, rAd-PEDV-S provides only partial protection against the highly virulent PEDV challenge, which was as effective as that of the commercial inactivated vaccine.

## 4. Discussion

In recent years, many studies have been conducted on PEDV vaccines, including PEDV inactivated/killed vaccines, attenuated vaccines, and gene-engineering vaccines [14, 16]. However, to date, PEDV commercial vaccines cannot completely prevent and control PEDV infection, so it is still necessary to develop new vaccines and to improve current PEDV vaccine efficacy. Currently, recombinant live vector vaccines are a new hot research topic in the development of vaccines and have been widely applied in many human and animal vaccine development attempts [31, 32]. In the recent work on development of a PEDV vaccine, several recombinant live vector vaccines based on the PEDV S protein were constructed successively [19, 22, 23]. These recombinant live vector vaccines both elicited protective immunity against the PEDV challenge in piglets and demonstrated the potential of recombinant live vectors as a vaccine delivery platform capable of eliciting passive immunity against PEDV. Therefore, in this study, a recombinant adenovirus expressing the spike protein of genotype GIIa PEDV was constructed successfully, and its immunogenicity and protective efficiency as a vaccine candidate were evaluated in 4-week-old pigs.

The coding sequence of the S protein used in this study was derived from the highly virulent Chinese genotype GIIa PEDV strain CH/HBXT/2018 (GenBank accession number MH816969), which was isolated by our laboratory and propagated in Vero cells. The strain has high pathogenicity in piglets, with a high mortality of almost 100% in infected suckling piglets, and causes a serious clinical disease with symptoms such as watery diarrhea [28]. An antigenic analysis showed that the S1 region, especially within the NTD, was highly homologous with that of current field strains. Based on these characteristics, the strain CH/HBXT/2018 was selected as a candidate for use in the development of a novel PEDV vaccine.

Although the adenovirus-expressing vectors show more advantages than other vectors, such as easy construction, higher efficiency of gene transfer, higher titers, and strong induction of humoral, mucosal, and cellular immune responses, the strong immune responses induced by adenovirus proteins result in poor vaccine efficacy [25, 27]. The same result can also be found in this study. Although rAd-PEDV-S could induce high levels of specific antibodies, its efficacy was limited. Therefore, future studies should be carried out to further improve this vaccine candidate, such as increasing the immune dose, reducing the side effects of the adenovirus vector, and selecting the appropriate immunization route.

It is a well-known fact that anti-PEDV-specific IgA antibodies are thought to play an important role in protection because passive lactogenic immunity remains the most promising and effective way to protect neonatal suckling piglets from enteric diseases such as PEDV [8]. Protecting suckling piglets through lactogenic immunity is dependent on the gut-mammary-sIgA axis [8]. The results here showed that there is a good correlation between the levels of serum IgG and IgA induced by rAd-PEDV-S and proved that the adenovirus vector construct is capable of eliciting humoral immunity and protection against PEDV infection in neonatal piglets. However, in the next study, rAd-PEDV-S should be used to immunize pregnant sows to determine whether it produces sIgA antibodies that are secreted in the colostrum and milk and ultimately transferred to suckling piglets (gut-mammary-sIgA axis).

In this study, pigs from all four groups were challenged orally with 1 × 10^5^ TCID_50_ highly virulent genotype GIIb PEDV strain CH/HNPJ/2017 at 28 dpv. After the challenge, typical clinical signs of PEDV infection were observed in all four groups from 1 to 7 dpi. The results demonstrated that rAd-PEDV-S and the inactivated vaccine could markedly delay clinical signs, reduce virus shedding, and provide partial protection against the highly virulent PEDV challenge. The possible reasons for the low protective efficacy of rAd-PEDV-S and the inactivated vaccine in pigs are as follows: the inoculation dose in pigs in the challenge study was relatively higher than that in most of the previous PEDV animal experiments. Generally, most PEDV animal experiments use 100 TCID_50_ virus as an inoculum. However, here, 1 × 10^5^ TCID_50_ highly virulent genotype GIIb PEDV strain was used as the inoculum in the pig challenge study. Although our previous study determined that the median pig diarrhea dose (PDD_50_) of the challenge strain CH/HNPJ/2017 was 8.63 log_10_ PDD_50_/3 mL at 7 dpi, the experimental animals were 4-day-old conventional suckling pigs [28]. Therefore, the challenge inoculum dose in the present study cannot be completely in accordance with the previous PDD_50_. The results of this study indicated that a suitable inoculum dose is very important for a PEDV infection study and should be determined in advance in various ages of pigs.

In conclusion, the present study showed that the PEDV S protein derived from emerging PEDV variants could be inserted into an adenovirus vector and successfully expressed in vitro. Further animal studies showed that rAd-PEDV-S could induce a high humoral immune response and confer partial protection for pigs against the highly virulent PEDV challenge.

## Figures and Tables

**Table 1:** Experimental design of pig vaccination and challenge.

Experimental group^a^	Inoculum	Immunization route	Vaccination days	Challenge	
				Inoculum^e^	Dpv
G1	Ad-blank^b^	IM	0, 21	HNPJ	28
G2	rAd-PEDV-S^c^	IM	0, 21	HNPJ	28
G3	Inactivated vaccine^d^	IM	0, 21	HNPJ	28
G4	PBS	IM	0, 21	HNPJ	28

^a^Each group contained five 4-week-old weaned piglets. ^b–d^Pigs in these three groups were vaccinated intramuscularly with 10^10^ PFU/mL Ad-blank, 10^10^ PFU/mL rAd-PEDV-S, and 2 mL of commercial PEDV inactivated vaccine (inactivated virus of the genotype GIIb strain; virus titer before inactivation ≥ 10^7^ TCID_50_), respectively. ^e^Each pig was challenged orally with a virus suspension containing 2 × 10^5^ TCID_50_ highly virulent genotype GIIb PEDV strain CH/HNPJ/2017. Dpv: days post vaccination; IM: intramuscularly.

**Table 2:** Summary of clinical scores and fecal viral shedding for the challenged piglets in each group.

Dpi	Ad-blank^a^ (*n* = 5)			rAd-PEDV-S^a^ (*n* = 5)			Inactivated vaccine^a^ (*n* = 5)			PBS^a^ (*n* = 5)		
	NP	CT	CS	NP	CT	CS	NP	CT	CS	NP	CT	CS
0	0/5	—	0	0/5	—	0	0/5	—	0	0/5	—	0
1	1/5	25.67	2	0/5	—	0	0/5	—	0	2/5	23.42–26.79	3
2	3/5	22.36–23.82	2‐3	0/5	—	0	0/5	—	0	5/5	22.73–25.18	3
3	5/5	21.58–24.12	2‐3	0/5	—	0	0/5	—	0			
4				1/5	23.02	2	1/5	25.44	1			
5				2/5	21.52–26.26	2	2/5	24.58–26.43	3			
6				3/5	23.54–28.09	2‐3	2/5	23.69–26.87	2			
7				3/5	24.27–25.59	2‐3	2/5	25.80–28.99	2‐3			

^a^Pigs were challenged orally with 2 mL of 1 × 10^5^ TCID_50_ highly virulent genotype GIIb PEDV strain. Dpi: days post inoculation for pigs; NP: number of PEDV-positive piglets; CT: cycle threshold value—a value greater than 30 was considered negative or below the detection limit of real-time PCR; CS: clinical score for fecal consistency—0 = normal, 1 = pasty, 2 = semiliquid, and 3 = liquid; —: samples with no CT value (no PEDV RNA detected). The blocks without any entry indicate that the PEDV-positive piglets were euthanized after infection was confirmed by clinical symptoms and fecal viral shedding.

## Data Availability

The data used to support the findings of this study are available from the corresponding author upon request.
